# Left subclavian artery revascularization in thoracic endovascular aortic repair: single center’s clinical experiences from 171 patients

**DOI:** 10.1186/s13019-021-01593-w

**Published:** 2021-07-30

**Authors:** Wei Xie, Yunxing Xue, Shuchun Li, Min Jin, Qing Zhou, Dongjin Wang

**Affiliations:** 1 Department of Cardiothoracic Surgery, Nanjing Drum Tower Hospital, The Affiliated Hospital of Nanjing University Medical School, 321 Zhongshan Road, Nanjing, 210000 Jiangsu People’s Republic of China; 2 Nanjing Drum Tower Hospital Clinical College of Nanjing Medical University, Nanjing, People’s Republic of China; 3 Institute of Cardiothoracic Vascular Disease, Nanjing University, Nanjing, People’s Republic of China

**Keywords:** Thoracic aortic disease, Thoracic endovascular aortic repair, Left subclavian artery, Revascularization

## Abstract

**Background:**

Left subclavian artery revascularization (LSA) is frequently performed in the setting of thoracic endovascular repair (TEVAR). The purpose of this study was to compare different techniques for LSA revascularization during TEVAR.

**Methods:**

We performed a single center’s retrospective cohort study from 2016 to 2019. Patients were categorized by LSA revascularization methods, including direct coverage without revascularization (Unrevascularized), carotid-subclavian bypass (CSB), fenestrated TEVAR (F-TEVAR). Indications, demographics, operation details, and outcomes were analyzed using standard statistical analysis.

**Results:**

171 patients underwent TEVAR with LSA coverage, 16.4% (n = 28) were unrevascularized and the remaining patients underwent CSB (n = 100 [58.5%]) or F-TEVAR (n = 43 [25.1%]). Demographics were similar between the unrevascularized and revascularized groups, except for procedure urgent status (*p* = 0.005). The incidence of postoperative spinal cord ischemia was significantly higher between unrevascularized and revascularized group (10.7% vs. 1.4%; *p* = 0.032). There was no difference in 30-day and mid-term rates of mortality, stroke, and left upper extremity ischemia. CSB was more likely time-consuming than F-TEVAR [3.25 (2.83–4) vs. 2 (1.67–2.67) hours, *p* = 0], but there were no statistically significant differences in 30-day or midterm outcomes for CSB versus F-TEVAR. During a mean follow-up time of 24.8 months, estimates survival rates had no difference.

**Conclusions:**

LSA revascularization in zone 2 TEVAR is necessary which is associated with a low 30-day rate of spinal cord ischemia. When LSA revascularization is required during TEVAR, CSB and F-TEVAR are all safe and effective methods, and F-TEVAR appears to offer equivalent clinical outcomes as a less time-consuming and minimally invasive alternative.

## Background

Thoracic endovascular aortic repair (TEVAR) has gained widespread acceptance and serves as the predominant treatment approach for patients with thoracic aortic diseases. Durable outcomes of TEVAR require an adequate proximal seal zone. It is estimated that 40% of TEVAR will require coverage of zone 2 to create a proximal seal [1]. The coverage of the left subclavian artery during TEVAR to achieve a proximal seal is associated with increased risk of stroke, spinal cord ischemia, and upper extremity ischemia [2]. In 2009, the Society for Vascular Surgery consensus statement recommended routine revascularization of the left subclavian artery when covered by TEVAR for proximal sealing in elective cases. However, the statement noted that their recommendations were based on low-quality evidence [3]. Besides, the recommendations did not address the most effective technique for LSA revascularization. The purpose of this study was to compare 30-day and mid-term outcomes of LSA coverage during TEVAR and evaluate revascularization techniques for the LSA including carotid-subclavian bypass (CSB) and fenestrated thoracic endovascular aortic repair (F-TEVAR).

## Methods

### Patients

From January 2016 to December 2019, 171 patients underwent TEVAR with LSA coverage at Nanjing Drum Tower hospital, 16.4% (n = 28) were unrevascularized and the remaining patients underwent CSB (n = 100 [58.5%]) or F-TEVAR (n = 43 [25.1%]). Electronic medical records were reviewed to obtain data on patient demographics, periprocedural information, and associated outcome data.

All patients with thoracic aortic pathology aged 18–90 years were confirmed by CTA. Only those undergoing TEVAR for pathology requiring intentional coverage of the left subclavian artery to obtain a proximal seal were included. Both elective and emergent cases were included in the sample. The comparison groups were designated by exposure criteria. According to LSA revascularization methods, patients were divided into 3 groups, including Unrevascularized group, CSB group, F-TEVAR group. The first comparison group was unrevascularized LSA versus revascularized LSA. The second group was based on revascularization types, CSB versus F-TEVAR.

### Preoperative imaging and revascularization decision

General principles of LSA revascularization include dominant left vertebral artery, presence of a patent left internal mammary artery to coronary artery bypass graft, occluded right vertebral artery, functioning left upper extremity arteriovenous access, long-segment thoracic aortic coverage. Thoracic aortic pathology was diagnosed by CT angiography. The proximal vertebral arteries were all imaged on CT angiography. However, preoperative imaging of cerebral anatomy was not performed routinely. Choice of revascularization technique was determined by patient anatomy and physician preference. All patients in revascularized group were performed TEVAR and concomitant LSA revascularization. F-TEVAR includes pre-fenestration and situ fenestration. The stent-grafts were individually modified to preserve the flow of LSA by surgeons. For some unrevascularized and CSB cases, proximal occlusion was performed via catheter-based embolization if intraoperative type II endoleak occurs.

### Surgical procedures

#### Carotid-subclavian bypass technique

**Fig. 1:**
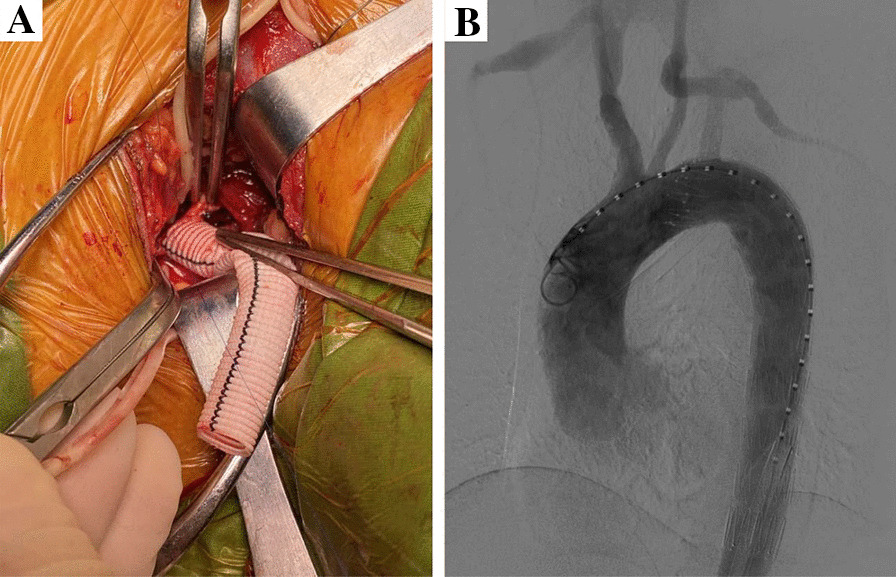
**A** Left common carotid artery-left subclavian artery bypass; **B** angiography showing left common carotid artery-left subclavian artery bypass graft patency and smooth left distal subclavian artery blood flow

Under general anesthesia, CSB was performed as the first stage of the procedure using standard supraclavicular incision and an 8-mm Dacron graft as bypass conduit (Fig. 1A). Then, TEVAR was performed with one femoral artery incised or directly punctured for access. The incision is usually packed and left open until placement of the TEVAR. The angiographic examination was used to ascertain treatment outcomes (Fig. 1B).

#### Situ fenestration technique

**Fig. 2:**
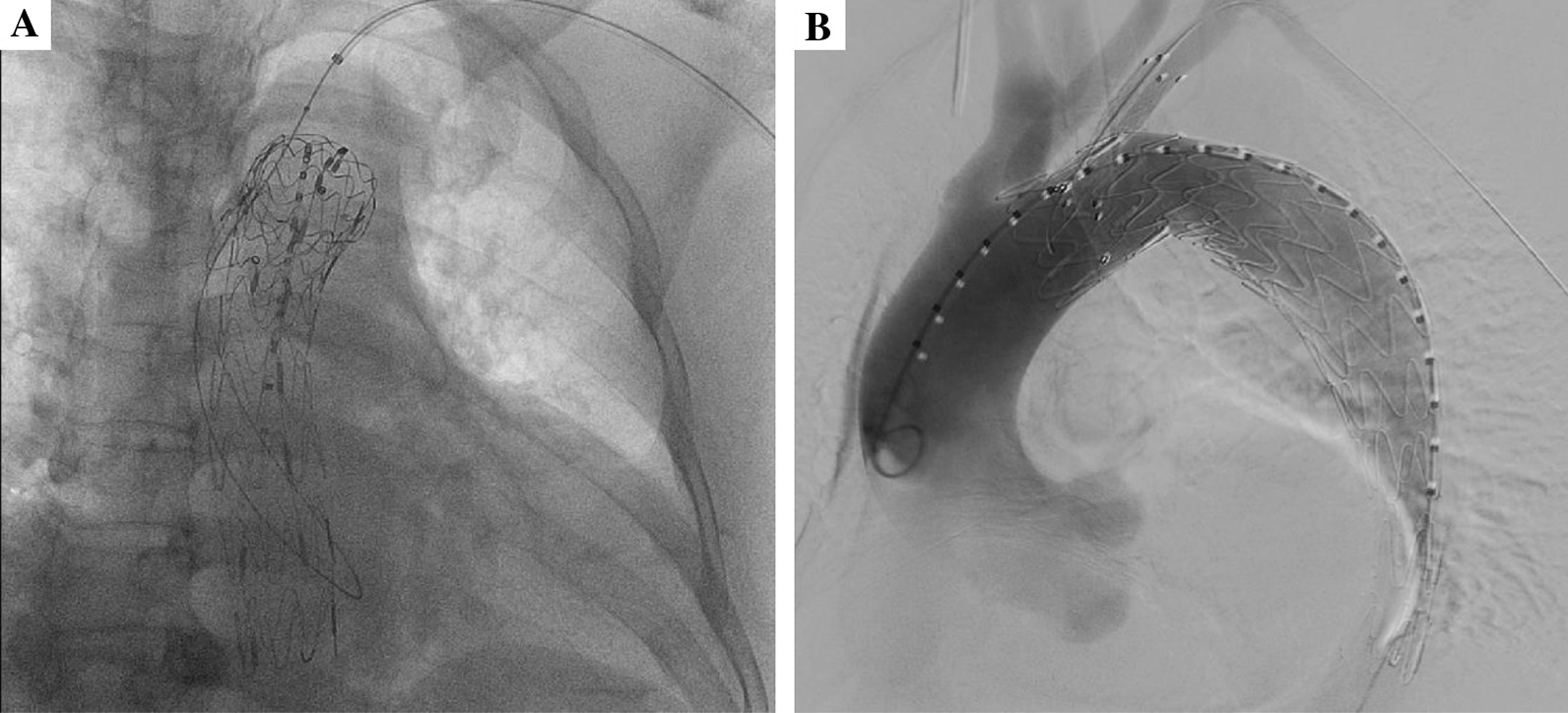
**A** In situ needle puncture is performed and a 0.014-inch guidewire was advanced through the aperture into the aorta; **B** DSA is repeated to confirm the exclusion of aortic disease and the patency of the LSA

Under general anesthesia, one femoral artery was exposed for the aortic stent grafts, and left brachial artery was then exposed for the fenestration of LSA. Then TEVAR was deployed with its proximal end next to the ostium of the LCCA. An 8F angle-adjustable sheath (Lifetech, Inc., Shanghai, China) was inserted retrograde from the left brachial artery until its tip reached the aortic stent graft. The tip was adjusted to be as perpendicular as possible to the greater curvature of the aortic stent graft. Once the sheath was located in the expected position, a flexible aspiration biopsy needle was used to penetrate the aortic stent graft to create the fenestrations. After puncturing, a 0.014-inch guidewire was advanced through the aperture into the ascending aorta (Fig. 2A). The initial aperture was then expanded by a 4-mm balloon, and then a 0.035-inch stiff guidewire was exchanged for 8-mm balloon expansion. Under these circumstances, self-expanding Fluency stent was implanted with its proximal end in the aortic stent graft. The angiographic examination was used to ascertain treatment outcome (Fig. 2B).

#### Pre-fenestration technique

**Fig. 3:**
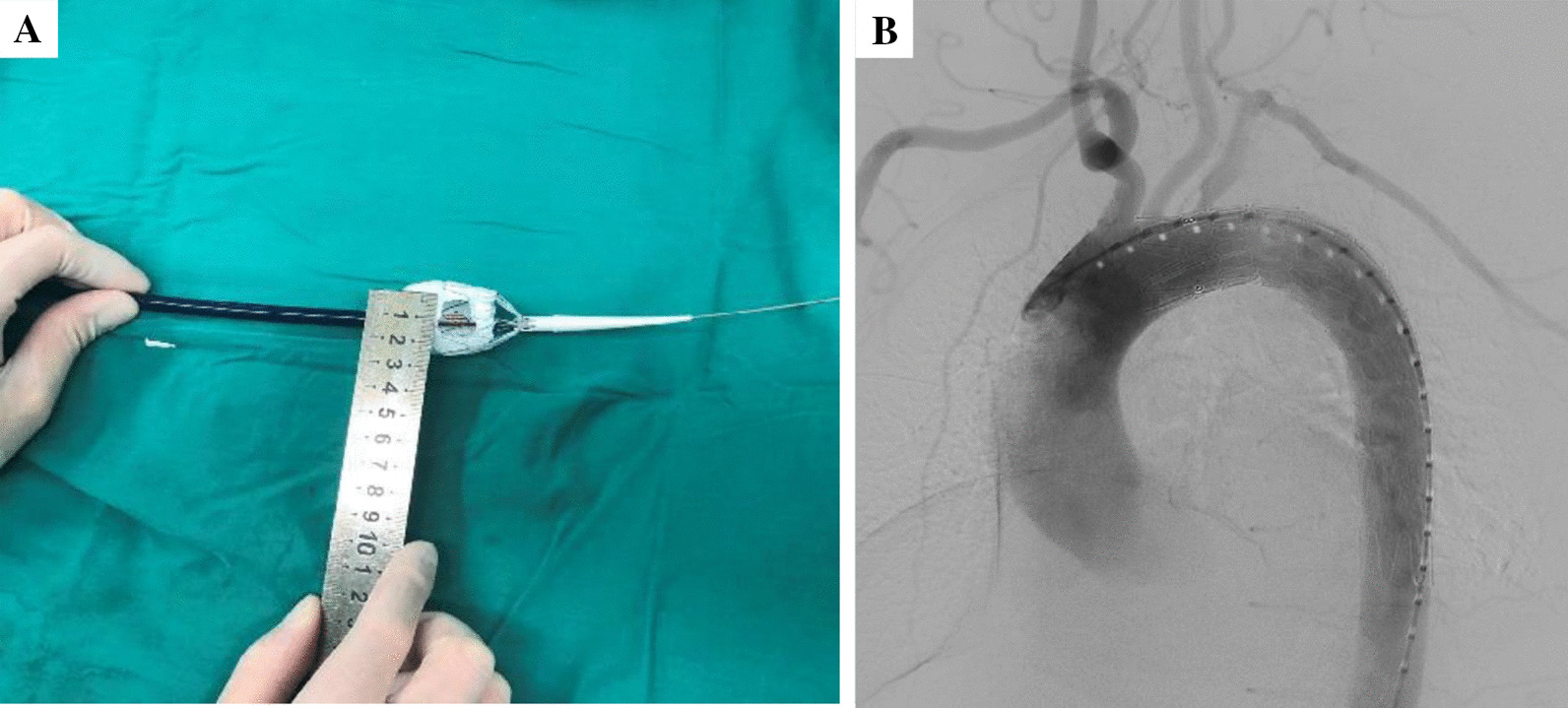
**A** The stent was released and underwent pre-fenestration; **B** DSA is repeated to confirm the treatment outcome and the patency of the LSA

Under general anesthesia, one femoral artery was incised or directly punctured for access; using CTA and intraprocedural angiography, the distance of the disease area from the LSA and the distal end of the LCCA, and the diameter of the proximal anchorage zone of the disease area, the diameter of the LSA. The stent was released and underwent pre-fenestration based on the latter measurements (Fig. 3A). Precise deployment of the fenestrated arch stent-grafts was confirmed by postprocedural angiography, and angiographic examination was used to ascertain treatment outcome (Fig. 3B).

### Outcomes

The outcomes of interest were mortality, stroke, left upper extremity ischemia, spinal cord ischemia (SCI). All were assessed clinically within 30 days after TEVAR and at midterm follow-up. The latter was defined as > 30 days and < 5 years. Stroke was defined as any new global or focal neurologic deficit lasting for > 24 h with an acute lesion on brain imaging [4]. SCI was defined as any new lower extremity deficit that was unrelated to an intracerebral event [4]. Left upper extremity ischemia was defined as weakness or numbness of the left upper extremity. All revascularized patients were treated with strict antiplatelet therapy for at least 3 months postoperatively. Most patients underwent postoperative surveillance imaging at 3 months, 6 months, and then annually.

### Statistical analysis

Categorical variables were analyzed with Pearson χ2 test, continuity corrected χ2 test or Fisher’s exact test. Continuous variables with normal distributions are presented as mean, standard deviation, and comparisons are made using Student t-test with significance accepted at *p* < 0.05. Skews distributions continuous variables are summarized as median, quartiles, and comparisons are made using non-parametric test with significance accepted at *p* < 0.05. Survival analysis was performed to estimated all-cause mortality. Statistical analysis was carried out using SPSS 25 (SPSS, Inc., Chicago, IL, USA).

## Results

From 2016 to 2019, a total of 547 TEVARs were performed in our center, of which 171 (31.2%) required coverage of the LSA. The 171 patients with LSA coverage had a mean age of 56.8 years, and 86% (n = 147) were male. The LSA was unrevascularized in 16.3% (n = 28) and revascularized in 83.7% (n = 143). The revascularized group included 69.9% CSB (n = 100) and 30.1% F-TEVAR (n = 43). Indications for TEVAR included dissection in 84.8% (n = 145), aneurysm in 5.8% (n = 10), penetrating aortic ulcers in 4.1% (n = 7), intramural hematoma in 5.3% (n = 9), respectively.

Demographics were similar between the unrevascularized and revascularized groups, except for procedure urgency. The unrevascularized group was more likely to have undergone TEVAR for urgent or emergent cases (*p* = 0.005). Demographics were similar between CSB and F-TEVAR groups, except there was a predominance of males in CSB group (90% vs. 76.7%, *p* = 0.036) (Table 1).

**Table 1:** Demographics and characteristics of Patients, stratified by procedure

Variables	Overall	Unrevascularized (n = 28)	Revascularized (n = 143)		*P*_1_ value	*P*_2_ value
			CSB(n = 100)	F-TEVAR(n = 43)		
Age (years, Mean ± SD)	56.75 ± 12.08	61.20 ± 14.45	55.43 ± 11.86	58.40 ± 10.57	0.254	0.15
Gender (Male, N, %)	147, 86%	24, 85.7%	90, 90%	33, 76.7%	1	0.036*
BMI (Kg/m2, Mean ± SD)	25.58 ± 3.24	25.79 ± 2.79	25.68 ± 3.22	25.28 ± 3.52	0.763	0.523
HTN (N, %)	126, 73.7%	21, 75%	73, 72.0%	33, 76.7%	0.967	0.556
DM (N, %)	12, 7.0%	0, 0%	9, 9.0%	3, 7.0%	0.236	0.943
CAD (N, %)	5, 2.9%	1, 3.6%	2, 2.0%	2, 4.7%	1	0.742
Previous open cardiac surgery (N, %)	9, 5.3%	2, 7.1%	4, 4.0%	3, 7.0%	0.981	0.738
Stroke history (N, %)	13, 7.6%	3, 10.7%	9, 9.0%	1, 2.3%	0.772	0.281
Chronic bronchitis (N, %)	4, 2.3%	1, 3.6%	3, 3.0%	0, 0%	0.514	0.554
CKD (N, %)	12, 7.0%	2, 7.1%	4, 4.0%	6, 14.0%	1	0.075
ESRD (N, %)	8, 4.7%	1, 3.6%	3, 3.0%	4, 9.3%	1	0.238
Smoking (N, %)	51, 29.8%	6, 21.4%	32, 32%	13, 30.2%	0.288	0.835
Hospital days (days, median, IQR)	18 (14–23)	16 (12.25–21)	19 (14–22.75)	18 (14–26)	0.195	0.711
Urgency					0.005*	0.521
Elective (> 24 h, N, %)	160, 93.6%	22, 78.6%	95, 95.0%	43, 100%		
Urgent (4–24 h, N, %)	8, 4.7%	4, 14.3%	4, 4.0%	0, 0%		
Emergent (< 4 h, N, %)	3, 1.8%	2, 7.1%	1, 1.0%	0, 0%		
Type of pathology (N, %)					0.084	0.607
Dissection	145, 84.8%	24, 85.7%	84, 84.0%	37, 86.0%		
Aneurysm	10, 5.8%	4, 14.3%	5, 5.0%	1, 2.3%		
PAU	7, 4.1%	0, 0%	6, 6.0%	1, 2.3%		
IMH	9, 5.3%	0	5, 5.0%	4, 9.3%		

*P*_1_ value, unrevascularied versus revascularized

*P*_2_ value, CSB versus F-TEVAR

BMI, body mass index; HTN, hypertension; DM, diabetes mellitus; AF, atrial fibrillation; CAD, coronary artery disease; CKD, chronic kidney disease; ESRD, end-stage renal disease; PAU, penetrating aortic ulcers; IMH, intramural hematoma

*Statistically significant values

Intraoperative outcomes were similar between two groups except for operation time (Table 2). Mean follow-up time was 24.8 months. Four patients lost follow-up. Thirty-day all-cause mortality was 0.6% (n = 1), and 6% (n = 10) at midterm for the sample. Strokes occurred in 6.4% (n = 11) of patients, of which, 18% (n = 2) patients occurred perioperatively, and 82% (n = 9) during follow-up. 1.2% (n = 2) of patients experienced the left upper extremity ischemia while in hospitalization, and 7.2% (n = 12) suffered the same symptom during follow-up. Spinal cord ischemia developed in 2.9% (n = 5) of patients during their hospitalization and there is no occurrence of spinal cord ischemia at follow-up. In addition, 1 patient underwent LSA embolization 2 weeks after TEVAR due to the occurrence of type II endoleak in unrevascularized group, and 1 patient needed to return to the operating room for incision bleeding in CSB group. During follow-up, 2 patients underwent delayed CSB for left upper extremity ischemia and dizziness four months after discharge from hospital in unrevascularized group, and 7 patients needed re-TEVAR to solve the aortic pathology such as endoleak or residual expanding false lumen.

**Table 2:** Operative Characteristics, stratified by procedure

Variables	Overall	Unrevascularized (n = 28)	Revascularized (n = 143)		*P*_1_ value	*P*_2_ value
			CSB (n = 100)	F-TEVAR (n = 43)		
Operation time (hours, median, IQR)	2.83 (2–3.67)	1.67 (1.5–2.5)	3.25 (2.83–4)	2 (1.67–2.67)	0*	0*
Right femoral access (N, %)	151, 88.3%	23, 82.1%	87, 87.0%	41, 95.3%	0.431	0.231
Number of stent (N, %)					0.877	0.261
One	139, 81.3%	24, 85.7%	77, 77%	38, 88.4%		
Two	29, 17.0%	4, 14.3%	20, 20%	5, 11.6%		
Three	3, 1.8%	0, 0%	3, 3%	0, 0%		
Cerebrospinal fluid drainage (N, %)	2, 1.2%	1, 3.6%	1, 1.0%	0, 0%	0.301	1
Vertebral originating from arch (N, %)	9, 5.3%	0, 0%	5, 5.0%	4, 9.3%	0.368	0.551
Coil embolization (N, %)	10, 5.8%	3, 10.7%	7, 7.0%	0, 0%	0.447	0.175

*P*_1_ value, unrevascularied versus revascularized

*P*_2_ value, CSB versus F-TEVAR

*Statistically significant values

**Fig. 4:**
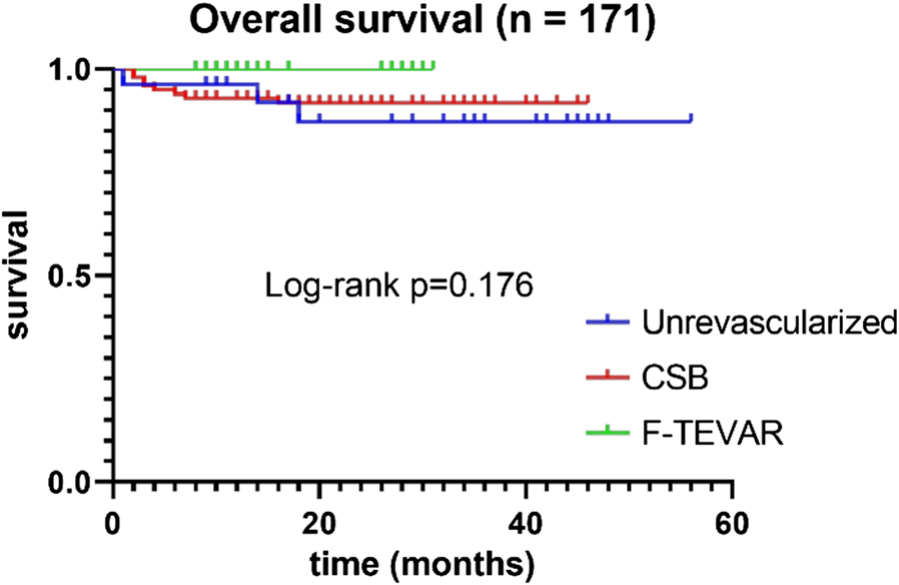
Kaplan–Meier estimates for survival, stratified by procedure

Comparisons between the unrevascularized and revascularized groups were significant for a higher rate of 30-day spinal cord ischemia (10.7% vs. 1.4%, *p* = 0.032). For the remaining outcomes, mortality, stroke, and left upper extremity ischemia were not statistically significant at 30-day or mid-term (Table 3). CSB was more likely time-consuming than F-TEVAR [3.25 (2.83–4) vs. 2 (1.67–2.67) hours, *p* = 0]. Comparison of 30-day and midterm outcomes, mortality, stroke, left upper extremity ischemia, and spinal cord ischemia for CSB versus F-TEVAR showed no statistically significant difference (Table 3). Comparison of 30-day and midterm overall study outcomes stratified by urgency showed no difference for stroke, left upper extremity ischemia, spinal cord ischemia, or mortality (Table 4). Kaplan–Meier estimates, stratified by procedure, were performed for survival. Survival estimates did not reach significance for unrevascularied LSA, CSB, or F-TEVAR (*p* = 0.176) (Fig. 4).

**Table 3:** Outcomes for Thoracic Endovascular Aortic Repair with Unrevascularized and Revascularized left subclavian artery, Carotid-Subclavian Bypass, and F-TEVAR

Variables	Overall	Unrevascularized (n = 28)	Revascularized (n = 143)		*P*_1_ value	iP_2_ value
			CSB (n = 100)	F-TEVAR (n = 43)		
30-day outcomes, n	171	28	100	43		
Mortality (N, %)	1, 0.6%	1, 3.6%	0, 0%	0, 0%	0.164	_
Stroke (N, %)	2, 1.2%	0, 0%	2, 2.0%	0, 0%	1	1
Left upper extremity ischemia (N, %)	2, 1.2%	0, 0%	2, 2%	0, 0%	1	1
Spinal cord ischemia (N, %)	5, 2.9%	3, 10.7%	1, 1.0%	1, 2.3%	0.032*	0.512
Midterm outcomes, n	167	26	100	41		
Mortality (N, %)	10, 6.0%	2, 7.7%	8, 8%	0, 0%	1	0.143
Stroke (N, %)	9, 5.4%	3, 11.5%	4, 4.0%	2, 4.9%	0.299	1
Left upper extremity ischemia (N, %)	12, 7.2%	4, 15.4%	6, 6.0%	2, 4.9%	0.177	1
Spinal cord ischemia (N, %)	0, 0%	0, 0%	0, 0%	0, 0%	_	_

*P*_1_ value, unrevascularied versus revascularized

*P*_2_ value, CSB versus F-TEVAR

*Statistically significant values

**Table 4:** Overall Study Outcomes, stratified by produce Urgency

Outcomes	Total (n = 171)	Elective (> 24 h) (n = 160)	Urgent (4–24 h) (n = 8)	Emergent (< 4 h) (n = 3)	*p* value
Mortality (N, %)	11, 6.4%	10, 6.3%	1, 12.5%	0, 0%	0.530
Stroke (N, %)	11, 6.4%	11, 6.9%	0, 0%	0, 0%	1.000
Left upper extremity ischemia (N, %)	14, 8.2%	14, 8.8%	0, 0%	0, 0%	1.000
Spinal cord ischemia (N, %)	5, 2.9%	4, 2.5%	0, 0%	1, 33.3%	0.103

## Discussion

Our study demonstrates there was a higher rate of 30-day SCI in patients with LSA coverage during TEVAR, and CSB and F-TEVAR were equivalent in terms of mortality, stroke, left upper extremity ischemia, and spinal cord ischemia at 30-day and mid-term.

There was a high rate of LSA unrevascularization in the urgently treated patients in our cohort. We performed selective revascularization in emergent cases and we were more likely to perform LSA revascularization in elective cases. For patients with acute thoracic emergency, TEVAR is required urgently and coverage of the LSA is necessary. LSA revascularization should be individualized and addressed on the basis of patient’s anatomy and urgency [5]. Revascularization of the left subclavian artery during TEVAR has led to various controversies among vascular groups and organizations initiating guideline recommendations [3, 6]. Nevertheless, there are no high-quality data to tip that revascularization is beneficial. Several studies are suggestive that revascularization is beneficial [1, 7, 8]. Other’s counter-argue that there are is no statistical differences [9–12]. Our study favors the former for the reason that there was a higher incidence of 30-day SCI in unrevascularized group. However, LSA coverage does not increase the incidence of mortality, stroke and left upper extremity ischemia in our study.

In those patients who underwent LSA origin coverage, the incidence of SCI was significantly higher in those patients who did not have surgical revascularization of the LSA (10.7% vs. 1.4%, *p* = 0.032). This result is consistent with previous studies [13, 14]. A report from the European Collaborators in Stent-Graft Techniques for Abdominal Aortic Aneurysm Repair (EUROSTAR) registry of 606 patients demonstrated an SCI rate of 2.5% and found coverage of the LSA without revascularization was an independent risk factor for SCI (OR, 3.9; *P* = 0.027) [13]. The risk factors for SCI have been reported including long segment coverage of thoracic aorta, LSA coverage, prior abdominal aortic repair, and perioperative hypotension, but length of aortic coverage is the only independent predictive factor of SCI after TEVAR [15]. This study did not describe precise length of aortic coverage, but the number of stents used is a reasonable surrogate for this and there is no difference in the number of stents between two groups.

Data from the Medtronic Thoracic Endovascular Registry (MOTHER) database suggested that coverage of the LSA without previous revascularization significantly increased the risk of stroke, most specifically in the posterior territory [7]. However, a large retrospective cohort that included patients with various aortic diseases failed to find any benefit in terms of postoperative stroke for prior selective LSA revascularization when covered during TEVAR [10]. Our study seems to support the latter. In the MOTHER database, patients with an aortic aneurysm and presumable more extensive atherosclerotic disease had a higher stroke rate compared with patients who had chronic dissection. It might be due to differences in patient selection and type of aortic disease inducing this difference outcomes. A recent meta-analysis demonstrated that revascularization of covered LSA in TEVAR is associated with significantly reduced risks of cerebrovascular accident, SCI, and left upper limb ischemia but not with increased risk of 30-day mortality [16]. Left subclavian revascularization theoretically avoids a low flow state of vertebral arteries which may induce stroke especially in those people whose Willis circle is intact, but it is difficult to accurately assess the integrality of Willis circle for all patients, especially in an emergency.

In our study cohort, comparisons between the unrevascularized and revascularized groups in left upper extremity ischemia were not statistically significant. Klocker et al. [17] investigated left arm ischemia and functional status and quality of life after LSA coverage during TEVAR using the Disabilities of the Arm, Shoulder, and Hand questionnaire and 12-Item Short Form Health Survey. Their results showed that TEVAR with LSA coverage is associated with low risk of left arm ischemia and no impact on left arm function and quality of life after the investigation. However, unrevascularization group had 2 patients need subsequent carotid-subclavian bypass for severe left arm ischemia which needed our attention.

Revascularization technique, CSB, and F-TEAVR did not reveal any significant difference in complication incidence in our sample. Thus, our findings would suggest that LSA revascularization during zone 2 TEVAR may be safely and effectively performed with either approach. Despite the increased arch manipulation and procedural complexity required for totally endovascular therapy of the LSA during zone 2 F-TEVAR, this may not translate into worse neurological outcomes. Besides, graft manipulation and the clamping of left common carotid could also increase rates of stroke in the CSB group [18]. Although unable to comment on the exact role of specific anatomic features in determining the prognosis of individual outcomes, the findings would suggest that, provided physician’s judgment of patients’ fitness for one technique (which is a complex decision based on the need to balance lowest possible perioperative morbidity in top of ensuring the longest durable outcomes possible), 30-day and midterm outcomes are equally satisfied with both CSB and F-TEVAR.

In our cohort, 30-day and midterm complications appeared to occur more commonly in CSB, but this difference was not statistically significant. It also might be argued that the additional open procedure might cause extra complications (particular local bleeding, nerve palsy, graft infection and lymphatic leak) [19, 20], while a totally endovascular approach to LSA revascularization would theoretically not be subject to these complications and would maintain the minimally invasive nature of TEVAR [21]. In our study, all patients underwent LSA fenestration during TEVAR were scheduled for elective surgery. CSB still serves as gold stand for late efficacy data of emerging endovascular solutions due to excellent patency rates and low rate of proximal landing zone reinterventions [19, 20]. Surgical subclavian bypass can be performed in urgent or even emergent cases and allow simpler exclusion of the aortic pathology by a more straightforward stent graft procedure [22]. Both situ fenestration and homemade fenestrated stent-graft need anatomic considerations. For situ fenestration, A 90° puncture or dilatation angle can avoid irregular fenestrations and fabric tears [23], which means more strict aortic arch morphology is required. For homemade fenestrated stent-graft, Graft rotation and misalignment of the vessel ostium interface can still occur [24]. Precise deployment of these fenestrated arch stent-grafts to correctly orient the fenestrations toward the branches for which they are intended also put higher demands on physicians. In addition, the durability of the stent-graft (Metal fatigue and material deterioration) after fenestration was a significant problem. Long-term monitoring of patients is required to avoid major complications resulting. In our series, no stent fractures were detected by follow-up radiologic examinations.

## Limitations

It was a retrospective study and the selection of the procedure lends itself to selection bias. The number of patients with fenestrated grafts increased with clinical experience. The unrevascularization groups accounting for only 16.3% of the entire study which may have influenced outcomes. Besides, the influence of aortic arch morphology on choice of surgical methods for the management of LSA needed further evaluation.

## Conclusions

Based on present study, LSA revascularization in zone 2 TEVAR is necessary and important which is associated a low 30-day rate of spinal cord ischemia. When LSA revascularization is required during TEVAR, CSB and F-TEVAR are all safe and effective methods, and F-TEVAR appears to offer equivalent clinical outcomes as a less time-consuming and minimally invasive alternative.

## Data Availability

The datasets generated and analyzed during the current study are available from the corresponding author on reasonable request.

## Abbreviations

TEVAR: thoracic endovascular aortic repair
LSA: left subclavian artery
LCCA: left common carotid artery
CSB: carotid-subclavian bypass
F-TEVAR: Fenestrated thoracic endovascular aortic repair
SCI: spinal cord ischemia
BMI: body mass index
HTN: hypertension
DM: diabetes mellitus
CAD: coronary artery disease
CKD: chronic kidney disease
ESRD: end-stage renal disease
PAU: penetrating aortic ulcers
IMH: intramural hematoma
RTAD: retrograde aortic dissection

## References

[1] Feezor RJ, Martin TD, Hess PJ, Klodell CT, Beaver TM, Huber TS, Seeger JM, Lee WA (2007). Risk factors for perioperative stroke during thoracic endovascular aortic repairs (TEVAR). J Endovasc Ther Off J Int Soc Endovasc Spec.

[2] Luehr M, Etz CD, Berezowski M, Nozdrzykowski M, Jerkku T, Peterss S, Borger MA, Czerny M, Banafsche R, Pichlmaier MA, Beyersdorf F, Hagl C, Schmidt A, Rylski B (2019). Outcomes after thoracic endovascular aortic repair with overstenting of the left subclavian artery. Ann Thorac Surg.

[3] Matsumura JS, Lee WA, Mitchell RS, Farber MA, Murad MH, Lumsden AB, Greenberg RK, Safi HJ, Fairman RM (2009). The society for vascular surgery practice guidelines: management of the left subclavian artery with thoracic endovascular aortic repair. J Vasc Surg.

[4] Lombardi JV, Hughes GC, Appoo JJ, Bavaria JE, Beck AW, Cambria RP, Charlton-Ouw K, Eslami MH, Kim KM, Leshnower BG, Maldonado T, Reece TB, Wang GJ (2020). Society for vascular surgery (SVS) and society of thoracic surgeons (STS) reporting standards for type B aortic dissections. Ann Thorac Surg.

[5] Upchurch GR, Escobar GA, Azizzadeh A, Beck AW, Conrad MF, Matsumura JS, Murad MH, Perry RJ, Singh MJ, Veeraswamy RK, Wang GJ (2021). Society for vascular surgery clinical practice guidelines of thoracic endovascular aortic repair for descending thoracic aortic aneurysms. J Vasc Surg.

[6] Riambau V, Böckler D, Brunkwall J, Cao P, Chiesa R, Coppi G, Czerny M, Fraedrich G, Haulon S, Jacobs MJ, Lachat ML, Moll FL, Setacci C, Taylor PR, Thompson M, Trimarchi S, Verhagen HJ, Verhoeven EL, Esvs Guidelines C, Kolh P, de Borst GJ, Chakfé N, Debus ES, Hinchliffe RJ, Kakkos S, Koncar I, Lindholt JS, Vega de Ceniga M, Vermassen F, Verzini F, Document R, Kolh P, Black JH, Busund R, Björck M, Dake M, Dick F, Eggebrecht H, Evangelista A, Grabenwöger M, Milner R, Naylor AR, Ricco JB, Rousseau H, Schmidli J (2017). Editor's choice: management of descending thoracic aorta diseases: clinical practice guidelines of the European Society for Vascular Surgery (ESVS). Eur J Vasc Endovasc Surg Off J Eur Soc Vasc Surg.

[7] Patterson BO, Holt PJ, Nienaber C, Fairman RM, Heijmen RH, Thompson MM (2014). Management of the left subclavian artery and neurologic complications after thoracic endovascular aortic repair. J Vasc Surg.

[8] Bradshaw RJ, Ahanchi SS, Powell O, Larion S, Brandt C, Soult MC, Panneton JM (2017). Left subclavian artery revascularization in zone 2 thoracic endovascular aortic repair is associated with lower stroke risk across all aortic diseases. J Vasc Surg.

[9] Sobocinski J, Patterson BO, Karthikesalingam A, Thompson MM (2016). The effect of left subclavian artery coverage in thoracic endovascular aortic repair. Ann Thorac Surg.

[10] Maldonado TS, Dexter D, Rockman CB, Veith FJ, Garg K, Arko F, Bertoni H, Ellozy S, Jordan W, Woo E (2013). Left subclavian artery coverage during thoracic endovascular aortic aneurysm repair does not mandate revascularization. J Vasc Surg.

[11] Hajibandeh S, Hajibandeh S, Antoniou SA, Torella F, Antoniou GA (2016). Meta-analysis of left subclavian artery coverage with and without revascularization in thoracic endovascular aortic repair. J Endovasc Ther Off J Int Soc Endovasc Spec.

[12] Janczak D, Ziomek A, Kobecki J, Malinowski M, Pormanczuk K, Chabowski M (2019). Neurological complications after thoracic endovascular aortic repair. Does the left subclavian artery coverage without revascularization increase the risk of neurological complications in patients after thoracic endovascular aortic repair?. J Cardiothorac Surg.

[13] Buth J, Harris PL, Hobo R, van Eps R, Cuypers P, Duijm L, Tielbeek X (2007). Neurologic complications associated with endovascular repair of thoracic aortic pathology: incidence and risk factors. A study from the European Collaborators on Stent/Graft Techniques for Aortic Aneurysm Repair (EUROSTAR) registry. J Vasc Surg.

[14] Cooper DG, Walsh SR, Sadat U, Noorani A, Hayes PD, Boyle JR (2009). Neurological complications after left subclavian artery coverage during thoracic endovascular aortic repair: a systematic review and meta-analysis. J Vasc Surg.

[15] Amabile P, Grisoli D, Giorgi R, Bartoli JM, Piquet P (2008). Incidence and determinants of spinal cord ischaemia in stent-graft repair of the thoracic aorta. Eur J Vasc Endovasc Surg Off J Eur Soc Vasc Surg.

[16] Chen X, Wang J, Premaratne S, Zhao J, Zhang WW (2019). Meta-analysis of the outcomes of revascularization after intentional coverage of the left subclavian artery for thoracic endovascular aortic repair. J Vasc Surg.

[17] Klocker J, Koell A, Erlmeier M, Goebel G, Jaschke W, Fraedrich G (2014). Ischemia and functional status of the left arm and quality of life after left subclavian artery coverage during stent grafting of thoracic aortic diseases. J Vasc Surg.

[18] Ramdon A, Patel R, Hnath J, Yeh C-C, Darling RC (2020). Chimney stent graft for left subclavian artery preservation during thoracic endograft placement. J Vasc Surg.

[19] Protack CD, Smith A, Moennich LA, Hardy D, Lyden SP, Farivar BS (2020). Midterm outcomes of subclavian artery revascularization in the setting of thoracic endovascular aortic repair. J Vasc Surg.

[20] Voigt SL, Bishawi M, Ranney D, Yerokun B, McCann RL, Hughes GC (2019). Outcomes of carotid-subclavian bypass performed in the setting of thoracic endovascular aortic repair. J Vasc Surg.

[21] D'Oria M, Karkkainen JM, Tenorio ER, Oderich GS, Mendes BC, Shuja F, Colglazier J, DeMartino RR (2020). Peri-operative outcomes of carotid-subclavian bypass or transposition versus endovascular techniques for left subclavian artery revascularization during non-traumatic zone 2 thoracic endovascular aortic repair in the Vascular Quality Initiative. Ann Vasc Surg.

[22] van der Weijde E, Saouti N, Vos JA, Tromp SC, Heijmen RH (2018). Surgical left subclavian artery revascularization for thoracic aortic stent grafting: a single-centre experience in 101 patients. Interact Cardiovasc Thorac Surg.

[23] Riga C, Bicknell C, Basra M, Hamady M, Cheshire NJ (2013). In vitro fenestration of aortic stent-grafts: implications of puncture methods for in situ fenestration durability. J Endovasc Ther.

[24] Canaud L, Morishita K, Gandet T, Sfeir J, Bommart S, Alric P, Mandelli M (2018). Homemade fenestrated stent-graft for thoracic endovascular aortic repair of zone 2 aortic lesions. J Thorac Cardiovasc Surg.

